# Food insecurity and health outcomes among community-dwelling middle-aged and older adults in India

**DOI:** 10.1038/s41598-023-28397-3

**Published:** 2023-01-20

**Authors:** Supa Pengpid, Karl Peltzer

**Affiliations:** 1 Department of Health Education and Behavioral Sciences, Faculty of Public Health, Mahidol University, Bangkok, Thailand; 2 Department of Public Health, Sefako Makgatho Health Sciences University, Pretoria, South Africa; 3 Department of Healthcare Administration, College of Medical and Health Science, Asia University, Taichung, Taiwan; 4 Department of Psychology, University of the Free State, Bloemfontein, South Africa; 5 Department of Psychology, College of Medical and Health Science, Asia University, Wufeng, Taichung, 41354 Taiwan

**Keywords:** Ecology, Risk factors

## Abstract

The study assessed associations between food insecurity and mental, physical, and behavioural health outcomes in India. The study analysed national cross-sectional population-based data (N = 72,262;  ≥ 45 years) from in India in 2017–2018. The overall prevalence of food insecurity was 9.7%. Food insecurity was significantly positively associated with poor mental health [low life satisfaction (AOR: 2.75, 95% CI 2.35–3.23), low self-reported health (AOR: 1.61, 95% CI 1.11–1.42), insomnia symptoms (AOR: 1.64, 95% CI 1.45–1.85), depressive symptoms (AOR: 2.21, 95% CI 1.97–2.48), major depressive disorder (AOR: 2.37, 95% CI 2.03–2.77), Alzheimer’s/dementia (AOR: 1.75, 95% CI 1.13–2.69), and poorer cognitive functioning (AOR: 0.68, 95% CI 0.49–0.93)], poor physical health [bone or joint disease (AOR: 1.18, 95% CI 1.04–1.34), angina (AOR: 1.80, 95% CI 1.58–2.06), underweight (AOR: 1.28, 95% CI 1.16–1.40), chronic lung disease (AOR: 1.22, 95% CI 1.03–1.45), and functional disability (AOR: 1.68, 95% CI 1.47–1.92)], and health risk behaviour [tobacco use (AOR: 1.13, 95% CI 1.01–1.25), heavy episodic drinking (AOR: 1.45, 95% CI 1.10–1.91) and physical inactivity (AOR: 1.42, 95% CI 1.21–1.67)]. Furthermore, food insecurity was negatively associated with overweight/obesity (AOR: 0.80, 95% CI 0.73–0.88). Food insecurity was associated with seven poor mental health indicators, five poor physical health conditions, and three health risk behaviours. Programmes and policies that improve food availability may help improve mental and physical health among middle-aged and older adults in India.

## Introduction

Globally, 25.9% of the population experienced hunger or had inadequate access to sufficient and nutritious food in 2019^1^. In India, for example, the prevalence of food insecurity was 8.5% in Delhi and Chennai^2^, in rural Puducherry, India, 31.7% had food insecurity^3^, and in a national sample of persons 50 years and older in India, 10.2% reported moderate food insecurity and 7.3% severe food insecurity^4^. Despite economic development, India has high food insecurity, hunger, malnutrition, undernourishment (16.3%) and underweight in children (34.4%)^5–7^. In India, the most populous nation to come, the population of older persons is globally growing faster than the general population to reach 34% of the total population by the end of the century^8^. Along with food insecurity various adverse health outcomes, including chronic diseases, poor mental health, disability, and reduced quality of life may be associated with ageing in India^8^.

Several reviews and large and small studies showed an association between food insecurity and poor mental health, such as depression^9–13^, mental morbidity^14^, anxiety^9^, not anxiety^10^, sleep disorder^9,10^, poorer subjective well-being^15^, and poorer cognitive function^11,16,17^. In terms of physical health outcomes, food insecurity has been associated with cardiometabolic risks, such as excess weight^18^, underweight^4^, hypertension^18,19^, self-reported hypertension^20^, cardiovascular diseases (angina, coronary heart disease, and heart attack)^19^, dyslipidaemias^18^, diabetes^18,21,22^, and physical frailty^23^.

Moreover, food insecurity has been associated with other physical chronic conditions, such as chronic lung disease^21^, inflammatory diseases or joint/muscular pain and functional limitations^24^. Regarding health risk behaviours, food insecurity and tobacco use were found to be bidirectionally associated^25^, while other studies found an association between food insecurity and physical inactivity and smoking^26^, heavy alcohol use, and smoking^27^.

However, studies investigation the association between food insecurity and poor mental health, poor physical conditions and health risk behaviours are usually conducted with one or two negative health outcomes and in high-income countries. To address, this shortfall, we aim to investigate the associations between food insecurity and a wide variety of health outcomes, including seven mental health, 11 physical conditions and three health risk behaviours, in a low-resourced country, India. To gain an understanding on associations between food insecurity with a wide range of health outcomes may assist in managing clients with food insecurity problems. Therefore, this study aimed to assess the associations between food insecurity and 21 health indicators in middle-aged and older adults in a national community-based study in India.

## Methods

### Study population and procedures

In a national cross-sectional household survey in India in 2017–2018, 72,262 individuals (≥ 45 years) and their spouses, regardless of age (response rate 87%) responded to a structured interview and physical measurements^29^. “Detailed information on sampling methods and sample size are published in the Longitudinal Ageing Study in India (LASI) Report^28^. The effective sample size for the present study was 72,262 middle-aged and older adults, including 41,685 males and 30,577 females. On the exposure variable food insecurity, 849 (1.2%) individuals were missing, which were excluded in the multivariable analysis, making the sample size 72,413. On the social and demographic variables (or control variables) the range of missing cases was from 0% for age, sex, and residence status to 1.0% for health insurance. The proportion of missing cases for the health outcome variables ranged from 0.3% for diagnosed hypertension and diabetes to 10.6% for body mass index. We compared missing with non-missing health outcome variables on social and demographic factors, and we could largely not find significant differences, except for older age and social participation with BMI and cognition, and no schooling, low organized religiosity, and rural residence with cognition. The study was approved by the “Indian Council of Medical Research (ICMR) Ethics Committee and written/oral informed consent was obtained from the participants”.

### Measures

#### Health outcome variables

Self-rated health status was defined as “1 = poor or fair and 0 = excellent, very good, or good”^28^; coded as in previous surveys in India^29^. Self‐rated health has been found to have high predictive validity for mortality^30^.

Life satisfaction was measured with the Health and Retirement Study item, “Please, think about your life as a whole. How satisfied are you with it?” “completely satisfied, very satisfied, somewhat satisfied (coded as 0), not very satisfied, or not at all satisfied (coded as 1)?”^31,32^.

Cognitive functioning was measured with four components (orientation, immediate and delayed word recall, and serial 7 s)^33^. These cognitive functioning tests have been previously validated in the Indian population^34–37^.

Insomnia symptoms (any of 4 items) were assessed with the “Jenkins Sleep Scale (JSS-4)”^38–40^ (Cronbach α 0.86).

Depressive symptoms (scores four or more) were obtained from the “Center for Epidemiological Studies Depression Scale (CES-D-10)”^41,42^. (Cronbach α was 0.79). The CES-D has been validated in the Indian population^42^, and in the Indian older adult population^43^.

Major depressive disorder (score ≥ 3, 0–7) was assessed with the “Composite International Diagnostic Interview Short Form (CIDI-SF)”^44,45^. CIDI-SF has been validated for use in general population health surveys^46^.

Anthropometry: “Height and weight of adults were measured using the Seca 803 digital scale.”^28^. The “body mass index = BMI was calculated according to Asian criteria: underweight (< 18.5 kg/m^2^), and overweight/obesity (≥ 23.0 kg/m^2^)”^47,48^.

Health care provider diagnosed “Alzheimer’s/Dementia,” “bone/joint disorder (arthritis/rheumatism, Osteoporosis/other bone/joint diseases),” “chronic heart diseases,” “Hypertension,” “diabetes,” “chronic lung disease” and “stroke” (Yes/No)^28^.

Angina was measured with the “World Health Organization’s Rose angina questionnaire,” defined on the basis of “discomfort at walking uphill or hurrying, or at an ordinary pace on level ground. Furthermore, the pain should be located at the sternum or in the left chest and arm, causing the patient to stop or slow down, and the pain should resolve within 10 min when the patient stops or slows down”^49–51^.

Elevated blood pressure (BP) or hypertension: “systolic BP ≥ 140 mm Hg and/or diastolic BP ≥ 90 mm Hg (based on the last two averaged of three readings) or on antihypertensive medication”^52^.

The difficulties of activities of daily living (ADL) (e.g. “Getting in or out of bed”) and instrumental activities of daily living (IADL) (e.g. “Taking medications”) were evaluated with 6 and 7 items, respectively (Yes, No)^53,54^; (Cronbach α was 0.89 in this study). Responses were dichotomized into “0 or 1 and ≥ 2 ADL and IADL items”. Both ADL and IADL measures have shown acceptable validity among older adults in India^55^.

Substance use included current heavy alcohol use (≥ 5 drinks on one occasion) and current tobacco use^28^.

Physical inactivity was defined as “hardly ever or never engaging in vigorous or moderate physical activity”^28^.

#### Exposure variable

LASI utilized four items of an 18-item Household Food Security Scale (HFSS)^56,57^, similar to items from the 8-item Food Insecurity Experience Scale Survey Module for Individuals (FIES SM-I)^58^. The HFSS items are validated in the Indian setting^59^. Both scales are to produce reliable food insecurity prevalence in diverse countries and use different scoring procedures, ranging from summative scores to individual scores^13,17^. The individual items are:

- (1) “In the last 12 months, did you reduce the size of your meals or skip meals because there was not enough food at your household? (Yes/No)”.
- (2) “In the last 12 months, were you hungry but didn’t eat because there was not enough food at your household? (Yes/No)”.
- (3) “In the past 12 months, did you ever not eat for a whole day because there was not enough food at your household? (Yes/No)”.
- (4) “Do you think that you have lost weight in the last 12 months because there was not enough food in your household? (Yes/No)”^57^.

The Cronbach α for the four-item HFSS was 0.82 in this study. Overall food insecurity was defined as 1–4 positive responses to four items.

### Covariates

Covariates consisted of sex, education, age, receipt of health insurance (“Are you covered by health insurance?”), marital and residential status, subjective economic status (1–3 = low, 4–5 = medium, and 6–10 = high, and caste (Scheduled tribes, scheduled castes, other backward classes, and none of these)^28^. Social participation [e.g. “Eat-out-of-house (restaurant/hotel)”] was defined as at least one of six daily to at least once a month social activity^60^. Organizational religiosity included frequency of attending religious services (“1 (low) = not at all, 2 (medium) = 1–3 times a month or ≥ 1 times/year, and 3(high) ≥ once a week”^28^.

### Data analysis

Considering the clustered study design, data analyses were performed with “STATA software version 15.0 (Stata Corporation, College Station, TX, USA).” Descriptive statistics were used for all participant characteristics and all outcome variables. Univariable and multivariable logistic and linear regressions were applied to assess the associations between food insecurity and binary and scale health outcomes (dependent variables). Variables significant in univariable analyses were included in the multivariable models. The health outcome variables were selected based on a previous review of the literature^9–27^. Multivariable models were adjusted for all social and demographic factors, and health variables. *P* < 0.05 was accepted as significant. The variance inflation factor (VIF) was calculated to check for multicollinearity, and none was found between the study variables.

### Ethical review

The study was approved by the “Indian Council of Medical Research (ICMR) Ethics Committee,” and written/oral informed consent was obtained from participants^28^.

### Ethics approval and consent to participate

The study was approved by the “Indian Council of Medical Research (ICMR) Ethics Committee in January 2017 and written or oral informed consent was obtained from the participants.” All methods were carried out in accordance with relevant guidelines and regulations and in accordance with the World Medical Association Declaration of Helsinki.

## Results

### Participant characteristics

The study analysed national cross-sectional population-based data (N = 72,262;  ≥ 45 years) from in India in 2017–2018. Most (75.1%) belonged to reserved castes or tribes, 49.5% had no formal education, 68.2% lived in rural areas, 20.7% had health insurance, 75.6% were married, 75.1% belonged to reserved castes / tribes, 68.2% lived in rural areas, 74.5% engaged in medium or high organised religiosity, and 54.4% had social participation. The prevalence of seven mental health indicators included, for example, 27.6% depressive symptoms and 0.7% Alzheimer’s/dementia, 11 physical health indicators included, for example, 46.1% overweight/obesity and 1.8% stroke, and three health risk behaviours, for example, 30.4% current tobacco use. The past 12-month prevalence of food insecurity was 9.7% (see Table 1).

**Table 1:** Sample and food insecurity characteristics among middle-aged and older adults in India, 2017–2018 (N = 72,262).

Variable	Variable specification	Sample	Food insecurity in the past 12 months				
			Overall (4 items)	Cut size/skip meals	Hungry but not eat	Not eat for whole day	Lost weight due to lack of food
		% or M (SD)	%	%	%	%	%
Social and demographic factors							
All			9.7	6.0	5.3	3.8	4.8
Age in years	45–59	54.1	9.1	5.6	5.0	3.4	4.1
	60 or more	45.9	10.5	6.4	5.6	4.2	5.7
Sex	Female	58.0	9.8	6.0	5.4	3.6	4.6
	Male	42.0	9.7	5.9	5.2	4.0	5.2
Formal education	1 or more years	50.5	7.3	4.2	3.8	2.5	3.4
	None	49.5	12.2	7.8	6.8	5.1	6.4
Socioeconomic status	Low	37.2	14.3	10.0	8.7	6.4	7.4
	Medium	38.7	8.1	4.3	4.0	2.8	3.6
	High	24.1	5.4	2.5	2.2	1.3	2.8
Health insurance	No	79.3	10.2	6.3	5.5	4.1	5.3
	Yes	20.7	8.0	4.7	4.8	2.5	3.0
Marital status	Not married	24.4	11.8	7.9	6.6	5.1	6.1
	Married	75.6	9.0	5.3	4.9	3.3	4.5
Religion	Hindu	81.9	9.3	5.7	5.0	3.7	4.8
	Muslim	11.7	12.7	7.3	7.1	4.6	6.3
	Christian	3.0	10.8	8.1	7.8	3.9	2.9
	Sikh	1.8	6.6	4.2	3.3	1.6	1.7
	Other	1.7	10.6	6.6	7.0	4.7	5.7
Caste/tribe	None of below	24.9	6.9	4.2	3.6	2.4	3.5
	Scheduled Caste	19.7	13.3	9.2	7.9	6.4	6.7
	Scheduled Tribe	8.8	11.2	7.3	6.0	4.3	5.6
	Other Backward Class	46.7	9.4	5.2	5.0	3.3	4.6
Residence	Rural	68.2	11.3	7.2	6.2	4.6	6.0
	Urban	31.8	6.3	3.3	3.3	2.0	2.4
Region	North	11.3	5.8	2.9	2.3	1.4	2.9
	Northeast	3.6	7.5	4.3	2.5	1.5	3.7
	East	23.4	10.9	7.9	6.6	4.5	5.8
	Central	20.8	13.7	7.8	6.4	5.6	9.4
	West	16.5	8.0	4.9	4.4	3.7	3.1
	South	24.4	8.4	5.0	5.6	2.9	2.3
Organised religiosity	Low	25.5	11.1	7.1	6.2	4.7	6.0
	Medium	46.9	9.2	5.7	5.1	3.5	4.5
	High	27.6	9.3	5.3	4.8	3.5	4.4
Social participation	Yes	54.4	9.7	5.7	5.3	3.5	4.5
Mental health							
Self-reported health (fair or poor)	Yes	39.7	13.0	8.3	7.8	5.2	6.8
Life satisfaction	Low	11.1	24.3	17.5	16.3	12.2	12.7
Cognitive functioning	Scores: 0–32; M (SD)	18.7 (5.1)	17.4 (5.1)	16.9 (5.1)	17.0 (5.2)	16.7 (4.8)	17.2 (5.0)
Insomnia symptoms	Yes	12.7	16.4	10.5	9.0	7.0	10.0
Depressive symptoms	Yes	27.6	16.9	11.2	9.9	7.2	9.3
Major depressive disorder	Yes	7.6	23.7	17.6	15.7	12.0	14.6
Alzheimer’s/dementia	Yes	0.7	18.4	12.9	11.8	8.5	10.9
Physical health							
General underweight	Yes	28.4	14.0	8.9	8.0	6.2	8.5
General overweight/obesity	Yes	46.1	6.8	4.1	3.4	2.4	2.6
Bone/joint disease	Yes	15.7	11.6	7.6	6.5	5.1	6.3
Hypertension (measured)	Yes	40.4	8.9	5.7	4.8	3.5	4.1
Hypertension (self-reported)	Yes	26.4	10.3	5.9	5.8	3.5	4.8
Heart disease	Yes	8.6	10.3	7.2	5.5	2.9	4.3
Angina	Yes	8.6	16.2	12.9	11.8	8.5	10.9
Stoke	Yes	1.8	15.8	10.4	8.6	4.3	8.7
Diabetes	Yes	11.6	8.2	3.8	4.7	2.4	3.4
Chronic lung disease	Yes	6.3	12.4	7.3	6.9	5.2	6.8
Functional limitations^a^	2 or more	28.8	13.8	9.0	7.9	5.8	7.4
Health risk behaviour							
Current tobacco use	Yes	30.4	11.5	7.6	6.6	5.1	6.3
Heavy alcohol use	Yes	2.9	15.5	10.3	10.1	5.0	5.9
Physical inactivity	Yes	23.7	11.8	6.5	5.7	4.2	6.6

^a^Difficulties with two or more Activities of Daily Living (ADL) and Instrumental Activities of Daily Living (IADL).

### Associations between food insecurity and health outcome indicators

In the fully adjusted multivariable models, food insecurity was significantly positively associated with poor mental health [low life satisfaction (AOR: 2.75, 95% CI 2.35–3.23), low self-reported health (AOR: 1.61, 95% CI 1.11–1.42), insomnia symptoms (AOR: 1.64, 95% CI 1.45–1.85), depressive symptoms (AOR: 2.21, 95% CI 1.97–2.48), major depressive disorder (AOR: 2.37, 95% CI 2.03–2.77), Alzheimer’s/dementia (AOR: 1.75, 95% CI 1.13–2.69), and poorer cognitive functioning (AOR: 0.68, 95% CI 0.49–0.93)], poor physical health [bone or joint disease (AOR: 1.18, 95% CI 1.04–1.34), angina (AOR: 1.80, 95% CI 1.58–2.06), underweight (AOR: 1.28, 95% CI 1.16–1.40), chronic lung disease (AOR: 1.22, 95% CI 1.03–1.45), and functional disability (AOR: 1.68, 95% CI 1.47–1.92)], and health risk behaviour [tobacco use (AOR: 1.13, 95% CI 1.01–1.25), heavy episodic drinking (AOR: 1.45, 95% CI 1.10–1.91) and physical inactivity (AOR: 1.42, 95% CI 1.21–1.67)]. Furthermore, food insecurity was negatively associated with overweight/obesity (AOR: 0.80, 95% CI 0.73–0.88). Food insecurity was not significantly associated with hypertension (self-reported and measured), heart disease, stroke, and diabetes. Compared to the summative food insecurity measure, similar associations were found when analysing each food insecurity item (cut size/skip meal, hungry but not eat, not eat for the whole day, and lost weight due to lack of food) separately (see Tables 2, 3, 4).

**Table 2:** Associations between food insecurity and mental health indicators.

Outcome variables	Response format	Food insecurity	Univariable odds ratio/exp (Coef.) (95% CI)	Multivariable odds ratio/exp (Coef.) (95% CI)^a^
Self-reported health (fair or poor)	No	Overall food insecurity	1 Reference	1 Reference
	Yes		1.82 (1.61 2.05)***	1.61 (1.11, 1.42)***
		Cut size/skip meals	1.95 (1.74, 2.20)***	1.64 (1.43, 1.87)***
		Hungry but not eat	2.19 (1.85, 2.61)***	1.93 (1.54, 2.41)***
		Not eat for whole day	1.90 (1.65, 2.19)***	1.58 (1.36, 1.84)***
		Lost weight due to lack of food	1.97 (1.75, 2.22)***	1.62 (1.43, 1.83)***
Life satisfaction (low)	No	Overall food insecurity	1 Reference	1 Reference
	Yes		3.75 (3.20, 4.40)***	2.75 (2.35, 3.23)***
		Cut size/skip meals	4.50 (3.83, 5.27)***	3.00 (2.51, 3.88)***
		Hungry but not eat	4.77 (3.83, 5.94)***	3.36 (2.74, 4.12)***
		Not eat for whole day	4.99 (4.14, 6.01)***	3.20 (2.61, 3.92)***
		Lost weight due to lack of food	3.66 (3.10, 4.31)***	2.49 (2.10, 2.96)***
Cognitive functioning	Scale	Overall food insecurity	1 Reference	1 Reference
			0.24 (0.15, 0.38)***	0.68 (0.49, 0.93)*
		Cut size/skip meals	0.16 (0.11, 0.23)***	0.54 (0.40, 0.74)***
		Hungry but not eat	0.17 (0.08, 0.38)***	0.52 (0.31, 0.87)*
		Not eat for whole day	0.13 (0.09, 0.19)***	0.52 (0.37, 0.72)***
		Lost weight due to lack of food	0.20 (0.14, 0.29)***	0.76 (0.54, 1.06)
Insomnia symptoms	No	Overall food insecurity	1 Reference	1 Reference
	Yes		2.04 (1.80, 2.32)***	1.64 (1.45, 1.85)***
		Cut size/skip meals	2.08 (1.80, 2.41)***	1.51 (1.33, 1.74)***
		Hungry but not eat	1.98 (1.66, 2.37)***	1.49 (1.27, 1.73)***
		Not eat for whole day	2.21 (1.83, 2.65)***	1.66 (1.37, 2.00)***
		Lost weight due to lack of food	2.60 (2.21, 3.06)***	1.98 (1.68, 2.35)***
Depressive symptoms	No	Overall food insecurity	1 Reference	1 Reference
	Yes		2.68 (2.32, 3.08)***	2.21 (1.97, 2.48)***
		Cut size/skip meals	3.04 (2.64, 3.50)***	2.31 (2.02, 2.64)***
		Hungry but not eat	2.96 (2.39, 3.67)***	2.31 (1.96, 2.72)***
		Not eat for whole day	3.08 (2.63, 3.61)***	2.29 (1.93, 2.71)***
		Lost weight due to lack of food	3.16 (2.73, 3.66)***	2.43 (2.08, 2.83)***
Major depressive disorder	No	Overall food insecurity	1 Reference	1 Reference
	Yes		2.90 (2.46, 3.41)***	2.37 (2.03, 2.77)***
		Cut size/skip meals	4.04 (3.34, 4.90)***	2.70 (2.28, 3.20)***
		Hungry but not eat	4.01 (3.15, 5.12)***	2.77 (2.29, 3.36)***
		Not eat for whole day	4.29 (3.54, 5.20)***	2.94 (2.38, 3.62)***
		Lost weight due to lack of food	4.09 (3.40, 4.92)***	2.70 (2.21, 3.29)***
Alzheimer’s/dementia	No	Overall food insecurity	1 Reference	1 Reference
	Yes		2.10 (1.47, 3.01)***	1.75 (1.13, 2.69)*
		Cut size/skip meals	2.36 (1.54, 3.62)***	1.99 (1.21, 3.29)**
		Hungry but not eat	2.40 (1.55, 3.72)***	1.98 (1.20, 3.29)**
		Not eat for whole day	2.40 (1.47, 3.91)***	2.24 (1.28, 3.94)**
		Lost weight due to lack of food	2.41 (1.58, 3.67)***	1.89 (1.12, 3.19)*

*CI*  confidence interval.

***p < 0.001, **p < 0.01, *p < 0.05.

^a^Adjusted for all social and demographic factors, and health variables.

**Table 3:** Associations between food insecurity and physical health indicators.

Outcome variables	Response format	Food insecurity	Univariable odds ratio (95% CI)	Multivariable odds ratio (95% CI)^a^
General underweight	No	Overall food insecurity	1 Reference	1 Reference
	Yes		1.78 (1.62, 1.95)***	1.28 (1.16, 1.40)***
		Cut size/skip meals	1.80 (1.61, 2.01)***	1.25 (1.11, 1.40)***
		Hungry but not eat	1.94 (1.71, 2.20)***	1.36 (1.19, 1.55)***
		Not eat for whole day	2.07 (1.81, 2.38)***	1.41 (1.22, 1.64)***
		Lost weight due to lack of food	2.27 (1.99, 2.59)***	1.51 (1.32, 1.73)***
General overweight/obesity	No	Overall food insecurity	1 Reference	1 Reference
	Yes		0.56 (0.51, 0.62)***	0.80 (0.73, 0.88)***
		Cut size/skip meals	0.54 (0.47, 0.61)***	0.80 (0.71, 0.91)***
		Hungry but not eat	0.53 (0.47, 0.60)***	0.81 (0.71, 0.92)***
		Not eat for whole day	0.51 (0.44, 0.58)***	0.79 (0.68, 0.92)**
		Lost weight due to lack of food	0.38 (0.33, 0.43)***	0.57 (0.50, 0.65)***
Bone/joint disease	No	Overall food insecurity	1 Reference	1 Reference
	Yes		1.27 (1.12, 1.44)***	1.18 (1.04, 1.34)*
		Cut size/skip meals	1.37 (1.18, 1.60)***	1.25 (1.07, 1.47)**
		Hungry but not eat	1.29 (1.08, 1.55)**	1.16 (0.97, 1.38)
		Not eat for whole day	1.47 (1.22, 1.78)***	1.29 (1.05, 1.58)*
		Lost weight due to lack of food	1.40 (1.18, 1.64)***	1.32 (1.10, 1.58)**
Hypertension (measured)	No	Overall food insecurity	1 Reference	1 Reference
	Yes		0.86 (0.78, 0.95)**	0.93 (0.85, 1.02)
		Cut size/skip meals	0.90 (0.80, 1.01)	0.97 (0.87, 1.09)
		Hungry but not eat	0.90 (0.79, 1.02)	0.95 (0.84, 1.08)
		Not eat for whole day	0.86 (0.75, 0.99)*	0.97 (0.84, 1.12)
		Lost weight due to lack of food	0.75 (0.67, 0.86)***	0.86 (0.76, 0.97)*
Hypertension (self-report)	No	Overall food insecurity	1 Reference	–
	Yes		1.09 (0.94, 1.26)	
		Cut size/skip meals	0.97 (0.84, 1.12)	
		Hungry but not eat	1.15 (0.89, 1.48)	
		Not eat for whole day	0.92 (0.78, 1.08)	
		Lost weight due to lack of food	0.98 (0.86, 1.12)	
Heart disease	No	Overall food insecurity	1 Reference	–
	Yes		1.07 (0.76, 1.49)	
		Cut size/skip meals	1.24 (0.79, 1.92)	
		Hungry but not eat	1.05 (0.62, 1.77)	
		Not eat for whole day	0.76 (0.48, 1.19)	
		Lost weight due to lack of food	0.88 (0.61, 1.27)	
Angina	No	Overall food insecurity	1 Reference	1 Reference
	Yes		1.93 (1.68, 2.23)***	1.80 (1.58, 2.06)***
		Cut size/skip meals	1.89 (1.59, 2.25)***	1.69 (1.42, 2.01)***
		Hungry but not eat	1.72 (1.40, 2.10)***	1.58 (1.31, 1.92)***
		Not eat for whole day	2.12 (1.76, 2.55)***	1.93 (1.60, 2.34)***
		Lost weight due to lack of food	2.19 (1.85, 2.58)***	1.96 (1.65, 2.32)***
Stroke	No	Overall food insecurity	1 Reference	1 Reference
	Yes		1.76 (1.15, 2.70)**	1.21 (0.90, 1.62)
		Cut size/skip meals	1.85 (1.02, 3.34)*	1.12 (0.79, 1.57)
		Hungry but not eat	1.69 (0.83, 3.42)	0.93 (0.62, 1.39)
		Not eat for whole day	1.17 (0.80, 1.70)	0.99 (0.64, 1.54)
		Lost weight due to lack of food	1.89 (1.39, 2.57)***	1.44 (0.96, 2.14)
Diabetes	No	Overall food insecurity	1 Reference	1 Reference
	Yes		0.80 (0.56, 1.16)	0.84 (0.72, 1.01)
		Cut size/skip meals	0.59 (0.48, 0.73)***	0.80 (0.65, 0.99)*
		Hungry but not eat	0.86 (0.46, 1.62)	0.78 (0.62, 1.02)
		Not eat for whole day	0.60 (0.46, 0.77)***	0.87 (0.67, 1.13)
		Lost weight due to lack of food	0.65 (0.53, 0.81)***	0.95 (0.75, 1.19)
Chronic lung disease	No	Overall food insecurity	1 Reference	1 Reference
	Yes		1.34 (1.10, 1.63)**	1.22 (1.03, 1.45)*
		Cut size/skip meals	1.27 (1.03, 1.55)*	1.11 (0.92, 1.34)
		Hungry but not eat	1.34 (1.02, 1.75)	1.21 (0.95, 1.54)
		Not eat for whole day	1.44 (1.06, 1.95)*	1.24 (0.92, 1.67)
		Lost weight due to lack of food	1.46 (1.13, 1.89)**	1.19 (0.92, 1.53)
Functional limitations^b^	No	Overall food insecurity	1 Reference	1 Reference
	Yes		1.84 (1.62, 2.08)***	1.68 (1.47, 1.92)***
		Cut size/skip meals	1.96 (1.74, 2.21)***	1.77 (1.54, 2.03)***
		Hungry but not eat	1.92 (1.60, 2.30)***	1.74 (1.45, 2.09)***
		Not eat for whole day	2.06 (1.79, 2.36)***	1.80 (1.56, 2.09)***
		Lost weight due to lack of food	2.02 (1.79, 2.28)***	1.77 (1.55, 2.03)***

*CI*  confidence interval.

***p < 0.001,**p < 0.01, *p < 0.05.

^a^Adjusted for all social and demographic factors, and health variables, ^b^Difficulties with two or more Activities of Daily Living (ADL) and/or Instrumental Activities of Daily Living (IADL).

**Table 4:** Associations between food insecurity and health risk behaviour.

Outcome variables	Response format	Food insecurity	Univariable odds ratio (95% CI)	Multivariable odds ratio (95% CI)^a^
Current tobacco use	No	Overall food insecurity	1 Reference	1 Reference
	Yes		1.32 (1.19, 1.47)***	1.13 (1.01, 1.25)*
		Cut size/skip meals	1.48 (1.32, 1.66)***	1.22 (1.07, 1.40)**
		Hungry but not eat	1.42 (1.22, 1.65)***	1.26 (1.09, 1.47)**
		Not eat for whole day	1.63 (1.41, 1.69)***	1.17 (0.97, 1.41)
		Lost weight due to lack of food	1.51 (1.34, 1.69)***	1.09 (0.95, 1.25)
Heavy alcohol use	No	Overall food insecurity	1 Reference	1 Reference
	Yes		1.74 (1.21, 2.51)**	1.45 (1.10, 1.91)**
		Cut size/skip meals	1.86 (1.12, 3.07)*	1.33 (0.92, 1.92)
		Hungry but not eat	2.07 (1.23, 3.47)**	1.48 (1.01, 2.16)*
		Not eat for whole day	1.36 (1.02, 1.82)*	1.20 (0.86, 1.67)
		Lost weight due to lack of food	1.23 (0.89, 1.71)	1.27 (0.88, 1.84)
Physical inactivity	No	Overall food insecurity	1 Reference	1 Reference
	Yes		1.33 (1.16, 1.53***	1.27 (1.12, 1.43)***
		Cut size/skip meals	1.12 (0.96, 1.31)	0.99 (0.86, 1.14)
		Hungry but not eat	1.09 (0.91, 1.31)	1.04 (0.89, 1.20)
		Not eat for whole day	1.18 (0.99, 1.40)	1.05 (0.88, 1.25)
		Lost weight due to lack of food	1.58 (1.37, 1.82)***	1.42 (1.21, 1.67)***

*CI* confidence interval.

***p < 0.001, **p < 0.01, *p < 0.05, ^a^Adjusted for all social and demographic factors, and health variables.

## Discussion

The study aimed to assess the associations between food insecurity and mental, physical, and behavioural health outcomes in India. Results show for the first time that food insecurity was significantly positively associated with seven poor mental health indicators (low life satisfaction, low self-reported health, insomnia symptoms, depressive symptoms, major depressive disorder, Alzheimer’s/dementia, and poorer cognitive functioning), five poor physical health conditions (bone/joint disease, angina, underweight, chronic lung disease, and functional disability), and three health risk behaviours (tobacco use, heavy alcohol use and physical inactivity).

Consistent with previous research^9–17^, we found largely strong associations between food insecurity and poor mental health indicators, the highest for low life satisfaction (2.75), followed by major depressive disorder (2.37), depressive symptoms (2.21), Alzheimer’s or dementia (1.75), insomnia symptoms (1.64), poorer self-reported health (1.61) and lower cognitive functioning (0.68). Possible mechanisms involved in the relationship between food insecurity and poor mental health, including cognitive functioning, include chronic stress triggered by food insecurity that increases the release of glucocorticoids that produce depressive symptoms and cognitive decline^11,61^. Since this was a cross-sectional study, we were unable to determine the direction of the relationship between food insecurity and poor mental health. Some studies suggest a bidirectional association between food insecurity and poor emotional health^62,63^. In addition to food insecurity leading to poor mental health, poor mental health may for example reduce income generating activities limiting the ability to purchase food and thus become food insecure^63^. Nutrition interventions by reducing food insecurity may positively impact on mental health^64^.

Regarding cardiometabolic risk factors, we found, in agreement with previous research^19^ that food insecurity was associated with angina and in unadjusted analysis with stroke, but not with overweight or obesity, hypertension, and diabetes, as previously found^18,19,21,22^. It is possible that food insecure individuals with angina and/or stroke have a reduced ability to handle difficult chronic conditions, which may increase the risk or worsen their cardiovascular disease^19^. Contrary to previous findings in high-income settings^18^, we found that food insecurity was protective against overweight or obesity, and, in agreement with a previous study in India^4^, that food insecurity was associated with underweight. In this study, 28.4% of the participants were underweight, emphasising the need to address undernutrition together with food insecurity. Furthermore, consistent with previous results^21,24^, this study showed an association between food insecurity and bone or joint diseases, chronic lung diseases, and functional limitations. It is possible that people with food insecurity were more likely to have chronic conditions, such as angina, bone or joint disease, and underweight, which may lead to a greater risk of functional limitation or disability^24^. Perhaps people with food insecurity eat less healthy foods (such as energy-dense foods) and this has an impact on the presence of chronic diseases^65^. Indian ageing adults with angina, bone or joint diseases, chronic lung diseases, and functional limitations could benefit from focused interventions addressing food insecurity^26^. On the other hand, the relationship between food insecurity and cardiovascular disease risk, including angina, may be bidirectional^19,66^, and having bone or joint diseases and/or functional disability may reduce accessibility to food thereby increasing food insecurity^67,68^.

Consistent with several previous investigations^26,27,69^, food insecurity was associated with tobacco use, heavy drinking, and physical inactivity in this study. Some research^70^ indicates that expenditure on tobacco and/or alcohol may reduce the food budget. The association between food insecurity and physical inactivity may be attributed to food insecurity leading to poor physical and/or mental health, which could result in physical inactivity^26^. Substance users may need to receive health education that quitting tobacco use and quitting or reducing alcohol use not only has health but also financial benefits and can improve food security, and on a population level food availability and substance use control should be promoted^70^. Furthermore, some research has shown a bidirectional association between food insecurity and tobacco use^25,71^, and similarly some cross-sectional studies showed an association between food insecurity and problem drinking^72^ and other studies found an association between problem drinking and food insecurity in men^27^, potentially suggesting a bidirectional association between food insecurity and problem drinking.

Considering the possible link between food insecurity and various negative health outcomes, food insecurity issues should be included in health care management, e.g. by including food security screenings as part of health assessment, followed by an appropriate intervention such as food access programmes^62^. In addition, long-term programmes to prevent food insecurity are needed, such as linking food insecure households to social services^17^.

### Strength and limitations of the study

The study utilized a large nationally representative sample of aging adults in India showing the associations between food insecurity and a wide range of mental, physical, and behavioural health outcomes in a middle-income context. Food insecurity was only assessed with four items and not a full scale, which would have allowed for assessing different degrees of food insecurity. The large sample size may introduce the possibility of type 1 error, and findings should be interpreted with this limitation. Furthermore, most measures were limited due to self-report and the cross-sectional study design hinders us from drawing causative conclusions. Moreover, there is the possibility of reverse causation, considering that various studies^25,62,63,71^ showed that poor health outcomes may cause food insecurity and food insecurity may cause poor health outcomes. Our cross-sectional data do not allow us to draw causal or directional conclusions, given that we were unable to totally remove the effect of reverse causality. However, we controlled our models for a wide range of covariates and health variables as confounders in the analyses. Longitudinal studies are needed to confirm or disconfirm the direction of our findings.

## Conclusions

One in ten individuals (≥ 45 years) in India had past 12-month food insecurity. Food insecurity was significantly positively associated with poor mental health (low life satisfaction, low self-reported health, insomnia symptoms, depressive symptoms, major depressive disorder, Alzheimer’s/dementia, and poorer cognitive functioning), poor physical health (bone or joint disease, angina, underweight, chronic lung disease, and functional disability), and health risk behaviour (tobacco use, heavy episodic drinking, and physical inactivity). Programmes and policies that improve food availability may help improve mental and physical health among middle-aged and older adults in India.

## Data Availability

The data are available at the The Gateway to Global Aging Data (www.g2aging.org).
